# A multidisciplinary approach to optimizing care of patients treated with alpelisib

**DOI:** 10.1016/j.breast.2021.12.016

**Published:** 2021-12-27

**Authors:** Hope S. Rugo, Mario E. Lacouture, Marcus D. Goncalves, Umesh Masharani, Matti S. Aapro, Joyce A. O'Shaughnessy

**Affiliations:** a Department of Medicine (Hematology/Oncology), University of California San Francisco Helen Diller Family Comprehensive Cancer Center, San Francisco, CA, USA; b Dermatology Service, Department of Medicine, Memorial Sloan Kettering Cancer Center, New York, NY, USA; c Division of Endocrinology, Weill Department of Medicine, Weill Cornell Medicine, New York, NY, USA; d Department of Medicine (Endocrinology), University of California San Francisco, San Francisco, CA, USA; e Department of Oncology, Genolier Cancer Centre, Clinique de Genolier, Genolier, Switzerland; f Baylor University Medical Center Texas Oncology, US Oncology, Dallas, TX, USA

**Keywords:** Alpelisib, Breast cancer, *PIK3CA*, AE management, AE, adverse events, BID, twice a day, eGFR, estimated glomerular filtration rate, FPG, fasting plasma glucose, FSBG, fingerstick blood glucose, GI, gastrointestinal, HbA1c, glycosylated hemoglobin, HER2, human epidermal growth factor receptor 2, HR, hazard ratio, HR+, hormone receptor-positive, IM, intramuscular, IV, intravenous, mTOR, mammalian target of rapamycin, OGTT, oral glucose tolerance test, PG, plasma glucose, PI3K, phosphatidylinositol-3-kinase, PI3Ki, phosphatidylinositol-3-kinase inhibitor, PI3Kα, phosphatidylinositol-3-kinase regulatory subunit alpha, SC, subcutaneous, SGLT2i, sodium-glucose co-transporter 2 inhibitor, TID, thrice daily

## Abstract

**Purpose:**

The oral, α-specific phosphatidylinositol-3-kinase (PI3Kα) inhibitor alpelisib is the first PI3K inhibitor approved for the treatment of advanced breast cancer. As alpelisib is a relatively new therapeutic option, specific guidance and a multidisciplinary approach are needed to provide optimal patient care. The primary objective of this manuscript is to provide comprehensive guidance on minimizing and managing adverse events (AEs) for patients with advanced breast cancer who are receiving alpelisib.

**Methods:**

Clinical studies, prescribing information, published literature, and relevant guidelines were reviewed to provide recommendations on the prevention and management of alpelisib-associated AEs.

**Results:**

The most common AEs associated with alpelisib in the phase 3 SOLAR-1 trial were hyperglycemia and rash (which are considered on-target effects of PI3Kα inhibition) and gastrointestinal AEs, including diarrhea, nausea, and decreased appetite. These AEs require regular monitoring, early recognition, and prompt initiation of appropriate treatment. In addition, there are effective strategies to reduce the onset and severity of frequently observed AEs—in particular, onset of hyperglycemia and rash may be reduced by lifestyle changes (such as reduced intake of carbohydrates and regular exercise) and antihistamine prophylaxis, respectively. To reduce risk of severe hyperglycemia, it is essential to achieve adequate glycemic control prior to initiation of alpelisib treatment.

**Conclusion:**

Overall, alpelisib-associated AEs are generally manageable with prompt recognition, regular monitoring, and appropriate intervention, preferably with a multidisciplinary approach.

## Highlights

• Alpelisib-associated AEs are generally manageable and reversible
• PI3K inhibitor-associated hyperglycemia is an on-target and transient event
• Use of prophylactic antihistamines may reduce onset and severity of rash
• Enhanced monitoring and management guidance results in fewer drug discontinuations
• Further studies are ongoing to optimize management of alpelisib-associated AEs

## 1 Introduction

### 1.1 The phosphatidylinositol-3-kinase (PI3K) pathway in breast cancer

The PI3K pathway is the most frequently dysregulated pathway implicated in the development of breast cancer [1]. The PI3Ks are a family of lipid kinases that composes a major intracellular signaling pathway that responds to extracellular stimuli, nutrients, hormones, and growth factors, and regulates cellular proliferation, differentiation, growth, and migration [[1], [2], [3]]. Overactivation of this pathway has been associated with oncogenesis and resistance to endocrine, human epidermal growth factor receptor 2 (HER2)-directed, and cytotoxic therapy in breast cancer [1,3].

Among the three classes of PI3Ks (I-III), class I PI3Ks are often abnormally activated in breast cancer [1,2,4]. Class IA PI3Ks form a heterodimer consisting of a regulatory subunit (p85) and a catalytic subunit (p110). The catalytic p110α, p110β, and p110δ subunits are encoded by *PIK3CA*, *PIK3CB*, and *PIK3CD*, respectively [[1], [2], [3]]. *PIK3CA* mutation is the most common alteration of this pathway linked to breast cancer, with ≥80% of mutations occurring within the helical (E542K and E545K) and kinase (H1047R) domains of p110α [1,[4], [5], [6]]. *PIK3CA* mutation has been reported in 20%–40% of breast cancers, but the incidence differs across breast cancer subtypes [5,7].

### 1.2 Alpelisib clinical trial results

Alpelisib is an α-selective PI3K inhibitor (PI3Ki) that inhibits p110α 50 times more potently than other isoforms [8,9]. The safety and efficacy of alpelisib in combination with fulvestrant were evaluated in the phase 3 SOLAR-1 trial of patients with *PIK3CA-*mutated, hormone receptor-positive (HR+), HER2– advanced breast cancer who had received prior endocrine therapy. In the *PIK3CA*-mutated cohort, longer median progression-free survival was observed in patients treated with alpelisib plus fulvestrant (n = 169) compared with placebo plus fulvestrant (n = 172) (11 mo vs 5.7 mo; hazard ratio [HR] 0.65, *P* < 0.001). The most common adverse events (AEs) reported in the safety population for alpelisib (n = 284) and placebo groups (n = 287) were hyperglycemia, diarrhea, nausea, decreased appetite, rash, or maculopapular rash. Serious AEs were reported by 99 (34.9%) alpelisib-treated patients and 48 (16.7%) placebo-treated patients. Dose interruption due to AEs occurred in 189 (66.5%) alpelisib-treated patients and 40 (13.9%) placebo-treated patients, whereas dose reductions due to AEs occurred in 164 (57.7%) alpelisib-treated patients and 13 (4.5%) placebo-treated patients. Permanent discontinuation due to AEs occurred in 71 alpelisib-treated patients (25%) and 12 placebo-treated patients (4.2%). The most common AEs leading to discontinuation of alpelisib were hyperglycemia (18 patients, 6.3%) and rash (9 patients, 3.2%) [8].

Alpelisib plus fulvestrant is indicated for the treatment of postmenopausal women, and men, with HR+, HER2–, *PIK3CA*-mutated, advanced or metastatic breast cancer following progression on or after an endocrine-based regimen [10]. Although AEs associated with PI3Kis are not unique, proactive management is critical to minimize their incidence and severity and allow patients to stay on treatment longer. Because alpelisib is a relatively new treatment, and many physicians have limited experience with PI3Kis, oncologists and other healthcare providers managing patients with breast cancer likely need further guidance in managing these AEs.

## 2 Incidence, etiology, and management of adverse events associated with alpelisib

### 2.1 Hyperglycemia

Due to the high risk of developing severe hyperglycemia and lack of clinical safety data available, patients with pre-existing or new diagnosis of diabetes should only initiate alpelisib after good glycemic control is achieved.

Under normal fed conditions, insulin acts via intracellular PI3K to suppress glucose production by the liver and enhance glucose uptake from the blood into adipose tissue and skeletal muscle, the major sites of glucose disposal [4,6,11]. In diabetes, absolute or relative insulin deficiency results in hyperglycemia. According to the American Diabetes Association, diabetes is diagnosed if patients have ≥1 of the following: fasting plasma glucose (FPG) ≥126 mg/dL (7.0 mmol/L), glycosylated hemoglobin (HbA1c) ≥6.5% (48 mmol/mol), 2-h plasma glucose (PG) ≥200 mg/dL (11.1 mmol/L) by oral glucose tolerance test (OGTT), or classic symptoms of hyperglycemia (polyuria, polydipsia) and a random PG ≥ 200 mg/dL (11.1 mmol/L). The cutoff values for prediabetes are FPG 100–125 mg/dL (5.6–6.9 mmol/L), HbA1c 5.7%–6.4% (39–47 mmol/mol), or 2-h PG during 75 g OGTT of 140–199 mg/dL (7.8–11.0 mmol/L) [12].

#### 2.1.1 Etiology and incidence

Phosphatidylinositol-3-kinase inhibitors block the intracellular action of insulin on the liver and skeletal muscle, thereby creating a transient state of insulin resistance [6]. Therefore, hyperglycemia is an expected “on-target” effect of PI3K inhibition, especially for inhibitors of PI3Kα, which plays a key role in glucose homeostasis [6,9]. It has been shown that inhibition of mammalian target of rapamycin (mTOR), which is downstream of PI3K, increases hepatic glycogen breakdown (and glucose release) and decreases glucose uptake into skeletal muscle [13].

Patients with β-cell dysfunction and insulin-resistance are at higher risk for PI3Ki-associated hyperglycemia [6]. Phosphatidylinositol-3-kinase–induced hyperglycemia tends to be temporary as it activates a feedback mechanism leading to higher circulating insulin levels, restoring glucose homeostasis. There is also preclinical evidence that hyperinsulinemia can lead to partial reactivation of PI3K signaling; hence, administering insulin may theoretically interfere with the therapeutic activity of alpelisib [4]. However, further research is needed as there are currently no clinical data relating endogenous insulin levels in alpelisib-treated patients with clinical outcomes.

**Fig. 1:**
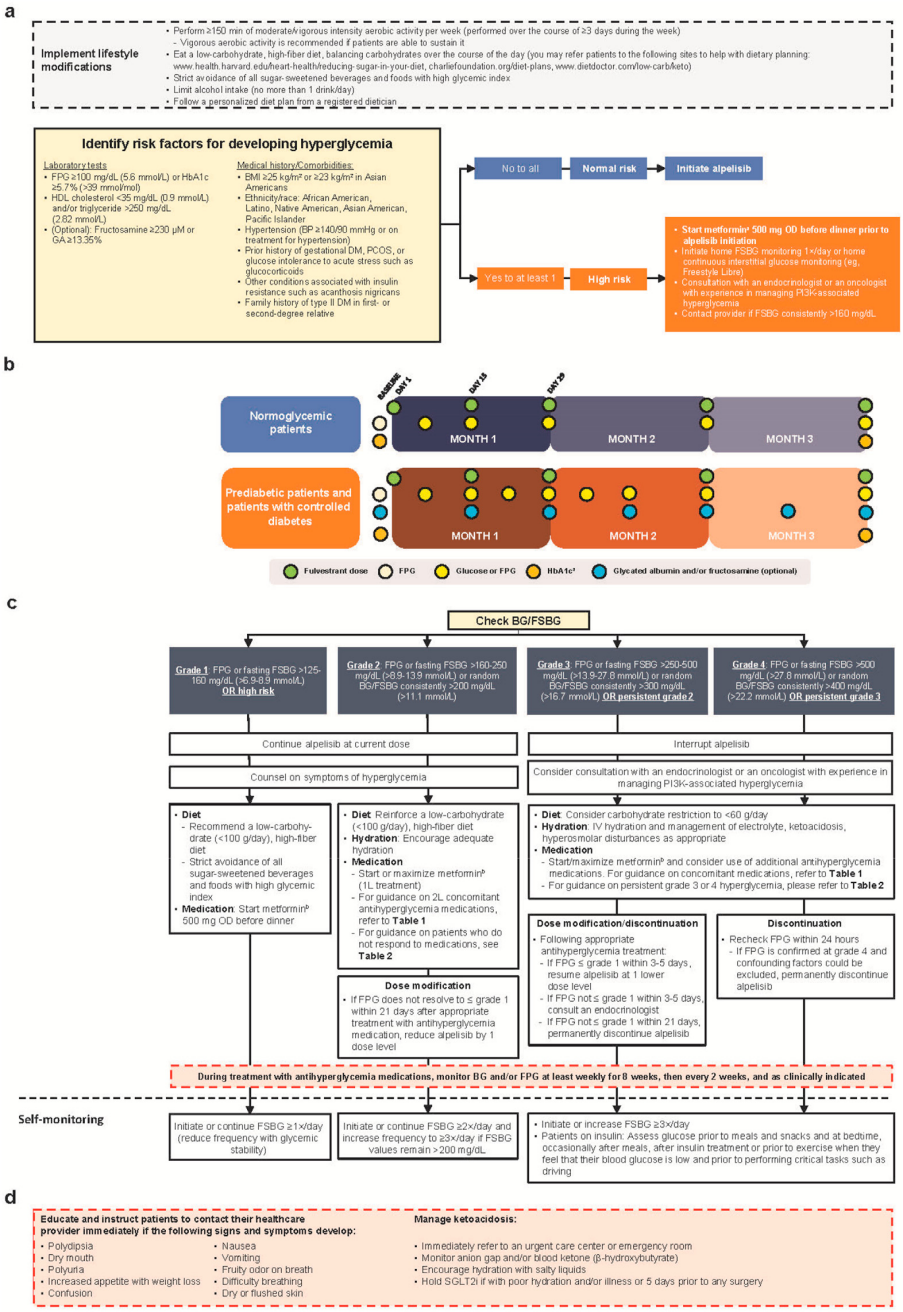
Management of alpelisib-associated hyperglycemia: [a] Lifestyle modifications and baseline glucose monitoring recommendations prior to initiating alpelisib treatment; [b] monitoring during alpelisib treatment; [c] hyperglycemia management recommendations; and self-monitoring guidelines during alpelisib treatment (severity based on CTCAE v4.03) [d] complications of hyperglycemia including ketoacidosis [10,12,[14], [15], [16]]. 1 L, first-line; 2 L, second-line; BG, blood glucose; BID, twice daily; BMI, body mass index; BP, blood pressure; CTCAE, Common Terminology Criteria for Adverse Events; DM, diabetes mellitus; eGFR, estimated glomerular filtration rate; FPG, fasting plasma glucose; FSBG, fingerstick blood glucose; GA, glycated albumin; HbA1c, glycosylated hemoglobin; HDL, high-density lipoprotein; IV, intravenous; OD, once daily; PCOS, polycystic ovarian syndrome; PI3K, phosphatidylinositol-3-kinase; SGLT2i, sodium-glucose co-transporter 2 inhibitor. ^a^May be done more frequently as clinically indicated. ^b^Assess eGFR prior to initiation of metformin; do not initiate metformin in patients with eGFR 30–45 mL/min/1.73 m^2^ but consider 50% dose reduction in patients already on metformin and monitor renal function every 3 months. Metformin is contraindicated in patients with eGFR <30 mL/min/1.73 m^2^ (see Table 1 [12,[15], [16], [17], [18], [19], [20], [21], [22], [23], [24], [25], [26], [27], [28], [29], [30], [31], [32], [33], [34], [35], [36], [37]] for further details) [18,19]. Initiate metformin at 500 mg OD before dinner, increasing to 500 mg BID (before breakfast and dinner) as tolerated. May increase to 500 mg at before breakfast then 1000 mg before dinner, and then to 1000 mg BID if tolerated. If not tolerated, reduce to prior tolerated dose.

Hyperglycemia at any grade was reported in 65% of alpelisib-treated patients, and 37% experienced grade ≥3 hyperglycemia (definitions of hyperglycemia grades found in Fig. 1). Median time to onset for grade ≥2 hyperglycemia was 15 days [10]. Treatment discontinuation was reported in 6.3% and 4.2% of alpelisib-treated patients due to any-grade and grade ≥3 hyperglycemia, respectively [8]. Identification of patients at risk and optimal management are therefore critical in maintaining therapeutic doses of alpelisib and avoiding unnecessary toxicity.

#### 2.1.2 Monitoring

Assess all patients for the risk of developing diabetes prior to initiating alpelisib, which includes thorough history taking, physical examination, and laboratory assessments as listed in Fig. 1a [12,14]. Optimization of blood glucose is necessary before initiating alpelisib treatment. Counsel patients on the symptoms of hyperglycemia. The safety of alpelisib in patients with diabetes mellitus type I or uncontrolled type II has not been established, as these patients were excluded from the SOLAR-1 and BYLieve trials [8,10,38]. In SOLAR-1, patients with HbA1c 6.5%–7.9% were initially allowed to enter the trial, but the study protocol was eventually amended to only include patients with HbA1c ≤ 6.5% and FPG ≤140 mg/dL. This was because patients with an HbA1c of 6.5%–8.0% were found to have an increased risk of developing grade ≥3 hyperglycemia [8]. Incidence of severe hyperglycemia (40.3% vs 32.9%) and discontinuation due to hyperglycemia (any grade: 9% vs 3.6%; grade 3/4: 5.6% vs 2.9%) were lower in the second half compared with the first half of patients randomized to alpelisib in SOLAR-1 [39]. This is likely due to protocol change and adjustments to monitoring and management of hyperglycemia. Overall, patients with HbA1c ≥ 6.5% at baseline should not initiate alpelisib until good glycemic control is achieved. However, patients with well-controlled type II diabetes (on medication) with HbA1c ≤ 7% at baseline may initiate alpelisib. Despite risk of PI3Ki-associated hyperglycemia, patients with controlled diabetes are already accustomed to managing blood sugar and may only require changes to treatment regimen. Encourage patients with prediabetes who are obese or overweight to lose weight (the American Diabetes Association recommends 7% loss of initial body weight). In patients who are able, encourage ≥150 min/week of moderate- to vigorous-intensity aerobic exercise spread over ≥3 days/week [12]. Advise patients to have a low-carbohydrate diet.

Closely monitor patients who are at risk for prediabetes and type II diabetes to allow early detection and prompt management of hyperglycemia, preferably in collaboration with an endocrinologist specializing in diabetic management or an oncologist with experience in treating patients with alpelisib [8]. One week prior to initiating alpelisib, patients at high risk should be instructed to do home glucose monitoring (fasting/random) with fingerstick blood glucose (FSBG) at least daily or home continuous interstitial glucose monitoring (eg, FreeStyle Libre monitoring system). Instruct patients to contact their healthcare provider for FSBG consistently >160 mg/dL. Glycated albumin and fructosamine are alternative markers of glycemia that can be monitored (usually by endocrinologists) at baseline and every 2 weeks for patients at high risk for diabetes [40]. Assess FPG and HbA1c at baseline for all patients. Only patients with baseline HbA1c <6.5% (≤7% if with pre-existing diabetes) should initiate alpelisib. Monitor blood glucose (fasting/random) at least once a week for the first 2 weeks, then at least once every 4 weeks, or at each visit, and as clinically indicated. Monitor HbA1c every 3 months, or as clinically indicated [10] (Fig. 1b) [12,14].

Individualize on-treatment glycemic targets based on prognosis and quality-of-life considerations. In healthier patients with a good prognosis, the recommended goals are premeal 90–130 mg/dL (5–7.2 mmol/L), 90–150 mg/dL (5–8.3 mmol/L) at bedtime, or HbA1c <7.5% (58 mmol/mol). Glycemic targets should be less stringent in patients who are frail and/or have poorer prognosis, as tight glycemic control may require excessive doses of antihyperglycemia medications or insulin. The recommended goals for those patients are premeal 100–180 mg/dL (5.6–10 mmol/L), 110–200 mg/dL (6.1–11.1 mmol/L) at bedtime, or HbA1c <8.5% (69 mmol/mol) [12]. If glycemic targets are not achieved, further action (including dose interruption and/or treatment discontinuation) may be required.

#### 2.1.3 Prevention and management

In addition to proper patient selection and appropriate medical management, the most powerful way to limit PI3Ki-associated hyperglycemia is to implement lifestyle changes that deplete hepatic glycogen. In animal models, hyperglycemia resulting from inhibition of the PI3K/protein kinase B pathway was mitigated by a period of fasting before the drug was dosed [41]. Fasting depletes hepatic glycogen; this deficit improved hyperglycemia in humans treated with other PI3K pathway inhibitors [41,42]. Hepatic glycogen starts to be depleted after 12 h of consecutive fasting (restricting food but not water) or with the application of a fasting-mimicking diet [43,44]. The ketogenic diet is similar to a fasting-mimicking diet due to its low carbohydrate composition but is high-fat and without calorie restriction [44]. Preclinical evidence demonstrated that ketogenic diet was able to reduce spikes in serum insulin and glucose and suppress downstream PI3K/mTOR signaling in PI3Ki-treated mice better than metformin, and also improve response to PI3Ki in multiple tumor types including breast cancer [4,45,46]. However, ketogenic diet is also associated with gastrointestinal (GI) symptoms, weight loss, and altered bowel habits [47]. We recommend ≥12 h (overnight) of daily consecutive fasting for patients who develop hyperglycemia while taking alpelisib. Alpelisib should be taken with food; hence, we recommend it be taken with a low-carbohydrate meal such as full-fat Greek yogurt (no sugar) or cheese omelet.

Instruct patients to follow American Diabetes Association or similar dietary guidelines. It is recommended that patients using insulin count carbohydrates. Limiting carbohydrates to <100 g can be helpful. Counsel patients on portion control and healthy food choices, with an emphasis on nutrient-dense and high-fiber foods including vegetables, fruits, whole grains, dairy, and legumes. Patients should strictly avoid sugar-sweetened beverages and rapid-release carbohydrate (high-glycemic-index) foods [12]. In patients with high-carbohydrate diet, it has been hypothesized that glucose is driven into tumors by hyperinsulinemia [4,45]. Consider referring diabetic patients to a dietician for individualized medical nutrition therapy [12]. Decreased appetite and weight were observed in clinical trials of alpelisib; hence, patients should be encouraged to maintain similar level of caloric intake [8,10,48].

Exercise should be recommended as it enhances the clearance of glucose from the blood into skeletal muscle via an insulin-dependent or an alternative, insulin-independent mechanism [49,50]. The latter is not expected to be hampered by PI3Ki and may be a viable way to reduce PI3Ki-associated hyperglycemia [50]. Furthermore, exercise improves insulin sensitivity, even in the setting of insulin resistance [49]. While patients are taking alpelisib, we recommend moderate- to vigorous-intensity aerobic exercise of ≥150 min/week spread over ≥3 days/week.

If lifestyle interventions are inadequate, manage with antihyperglycemia medications. Metformin is the preferred initial treatment option due to its wide availability and safety profile. Metformin suppresses hepatic gluconeogenesis, which is typically upregulated in patients with diabetes [51,52]. Assess estimated glomerular filtration rate (eGFR) prior to metformin initiation [18]. Do not initiate metformin if eGFR is <45 mL/min/1.73 m^2^, and discontinue or reduce dose by 50% if a patient's eGFR decreases to this level [19] (Table 1) [12,[15], [16], [17], [18], [19], [20], [21], [22], [23], [24], [25], [26], [27], [28], [29], [30], [31], [32], [33], [34], [35], [36], [37]]. When initiating metformin for prophylaxis or hyperglycemia management, use 500 mg once daily (before dinner) then titrate based on response and GI side effects up to a maximum of 1000 mg twice a day (BID). For grade ≤2 hyperglycemia, no alpelisib dose modification is needed. Late intervention (starting medication after 4 weeks or 3 weeks for grades 1 and 2, respectively) for grade 1/2 hyperglycemia resulted in a higher chance of hyperglycemia not improving or becoming severe [53]. If significant hyperglycemia develops, alpelisib dose reduction/interruption may lead to improved glucose control and stabilization. For grade ≥3 hyperglycemia, interrupt alpelisib treatment and consider consultation with an endocrinologist or an oncologist with experience in managing PI3Ki-associated hyperglycemia. If interrupting alpelisib, consider interrupting antihyperglycemia medication to avoid hypoglycemia (fulvestrant may be continued). Detailed guidance on hyperglycemia management and alpelisib dose interruption/modification is available in Fig. 1c [12,14].

If hyperglycemia is not controlled with metformin, add another antihyperglycemia medication (Table 2) [14]. Consider agents that do not affect the PI3K pathway, such as acarbose and sodium-glucose co-transporter 2 inhibitors (SGLT2is). In animal models of cancer, adding SGLT2i to a PI3Ki improved hyperglycemia and slowed tumor growth [46]. SGLT2is act on the kidneys to reduce the renal glucose threshold, leading to glucosuria, resulting in reduced plasma glucose and insulin levels [56]. Their use therefore will ameliorate the insulin feedback mechanism associated with PI3K inhibition. The combination of metformin and SGLT2is is safe and is widely used in treating patients with hyperglycemia, but this combination has not been formally tested in patients treated with alpelisib [57]. A case report described the development of euglycemic ketoacidosis (increased anion gap metabolic acidosis, ketonemia [>3 mM], or ketonuria [moderate to large on urinalysis], but with normal or modestly elevated blood glucose [<250 mg/dL or 13.9 mmol/L]) in a patient with breast cancer taking a PI3Ki with an SGLT2i [15,16,58]. Monitor anion gap and/or blood ketone (β-hydroxybutyrate) in patients treated with SGLT2is at each visit to assess for ketoacidosis. Counsel patients on the symptoms of ketoacidosis, including malaise, fatigue, nausea, and vomiting. Patients may be advised to measure ketones using a blood ketone meter (β-hydroxybutyrate goal <0.6–3.0 mmol/L) or urine (acetoacetate) testing [16]. Notably, nausea and vomiting are common alpelisib-associated AEs that may be difficult to distinguish from symptoms of ketoacidosis. Onset of ketoacidosis should prompt immediate referral to an urgent care center or emergency room.

**Table 1:** Regimens and characteristics of antihyperglycemia agents.

Class	Weight Loss [12,17]	Drug and Recommended Dosage [12]		Effect on Insulin [12,17]	Other Considerations for Treatment [12,17]
**Biguanide**	Yes	• Metformin 500–2000 mg PO BID		Decrease when glucose is low	• Assess eGFR prior to initiation of metformin; annual monitoring of renal function is recommended for eGFR ≥60 mL/min/1.73 m^2^ [18,19] • Monitor renal function every 3–6 months for eGFR 45–60 mL/min/1.73 m^2^ [19] • Do not initiate metformin in patients with eGFR 30–45 mL/min/1.73 m^2^; if already on metformin, discontinue metformin or consider 50% dose reduction and monitor renal function every 3 months [19] • Metformin is contraindicated in patients with eGFR <30 mL/min/1.73 m^2^ [18] • Risk of GI intolerance (bloating, abdominal discomfort, diarrhea), vitamin B12 deficiency, and lactic acidosis (rare)
**Secondary agents for hyperglycemia treatment** *For the management of grade ≥2 hyperglycemia in combination with metformin as needed*					
**SGLT2 inhibitors**	Yes	• Ertugliﬂozin 5–15 mg PO OD [20] • Dapagliﬂozin 5–10 mg OD [21] • Canagliﬂozin 100–300 mg AC OD [22] • Empagliﬂozin 10–25 mg OD [23]		Decrease when glucose is low	• Monitor for **euglycemic ketoacidosis** especially when patient is nauseous, vomiting, or dehydrated; encourage hydration with salty liquids; monitor anion gap and/or blood ketone (β-hydroxybutyrate) for patients treated with an SGLT2i at each visit to assess for ketoacidosis. An anion gap between 10 and 16 mEq/L is considered normal and >20 mEq/L indicates severe ketoacidosis [54,55]. Patients may be advised to measure ketones using a blood ketone meter (β-hydroxybutyrate goal 0.6–3.0 mmol/L) or urine (trace acetoacetate) testing. Hold SGLT2i if with poor hydration and/or illness (infection, etc) or 5 days prior to any surgery [15,16] • Associated with o Genitourinary infections o Increase in LDL cholesterol o Polyuria with risk of volume depletion and hypotension [24] o Glycosuria with risk of genitourinary infections including Fournier's gangrene in diabetics [24] o Additional risks (amputations, bone fractures) with canagliflozin treatment in diabetics
**GLP-1 receptor agonists**	Yes	• Exenatide (extended release) 2 mg SC once every 7 days [25] • Exenatide 5–10 μg/dose SC AC BID [26] • Dulaglutide 0.75–1.5 mg SC once weekly [27] • Semaglutide 3–14 mg PO AC OD or 0.25–1 mg SC once weekly [28,29] • Liraglutide 0.6–1.8 mg SC daily [30]		Decrease when glucose is low	• GI side effects are common (nausea, vomiting, diarrhea) • Injection-site reactions • Risk of pancreatitis • Risk of thyroid C-cell tumors
**DPP-4 inhibitors**	No	• Alogliptin 25 mg PO OD [31] • Saxagliptin 2.5–5 mg PO OD [32] • Linagliptin 5 mg PO OD [33] • Sitagliptin 100 mg PO OD [34]		Decrease when glucose is low	• Typically well tolerated • Potential risk of acute pancreatitis • Joint pain
**Thiazolidinediones**	No (chance of weight gain)	• Pioglitazone 15–45 mg PO OD [35] • Rosiglitazone 4–8 mg/day PO [36]		Decrease when glucose is low [36]	• Exercise extreme caution when using in patients with or at risk for congestive heart failure and patients at risk of falls/fractures
**α-Glucosidase inhibitors**	Yes	• Acarbose 25–100 mg TID with meals [37]		Decrease when glucose is low	• GI side effects (such as flatulence) are common
**Tertiary agents for hyperglycemia treatment** *For the management of grade ≥3 hyperglycemia in combination with metformin and secondary antihyperglycemia agents*					
**Sulfonylureas**	No	• Glimepiride 1–8 mg PO OD • Glipizide 5–40 mg PO (OD or split dose BID) • Glipizide XR 5–20 mg PO • Glyburide 2.5–20 mg OD • Glyburide micronized 3–12 mg OD		Increase	• Glyburide is contraindicated in patients with eGFR <30 mL/min/1.73 m^2^ • Risk of hypoglycemia, weight gain, diarrhea, nausea
**Meglitinides**	No	• Nateglinide 60–120 mg AC • Repaglinide 0.5–4 mg AC		Increase	• Risk of hypoglycemia, weight gain, diarrhea, nausea
**Insulin regimens** *For the management of persistent grade ≥3 hyperglycemia despite treatment with above agents*					
**Basal insulin** • Insulin glargine • Insulin detemir	No (chance of weight gain)	• 0.1–0.2 units/kg/day SC • Increase for goal fasting FSBG <160 mg/dL		Increase	• Risk of hypoglycemia, weight gain
**Rapid acting insulin** • Insulin aspart • Insulin lispro • Insulin glulisine	No (chance of weight gain)	**Sliding scale AC**		Increase	• Risk of hypoglycemia, weight gain
		**Pre-meal FSBG**	**Units**		
		70–99	0		
		100–149	0		
		150–199	2		
		200–249	3		
		250–299	4		
		300–349	5		
		350–399	6		
		>400	7		

AC, before meals; BID, twice daily; C-cells, parafollicular cells; DPP-4, dipeptidyl peptidase-4; eGFR, estimated glomerular filtration rate; FSBG, fingerstick blood glucose; GI, gastrointestinal; GLP-1, glucagon-like peptide; LDL, low-density lipoprotein; OD, once daily; SC, subcutaneous; SGLT2i, sodium-glucose co-transporter 2 inhibitor.

**Table 2:** Additional guidance for patients who do not respond to initial hyperglycemia treatment.

Hyperglycemia Severity (CTCAE v4.03) [14]	Initial Treatment Recommendations	Additional Treatment Recommendations	Recommended Alpelisib Dose Modifications
**Grade 2:** FPG or fasting FSBG >160–250 mg/dL (>8.9–13.9 mmol/L) or random BG/FSBG consistently >200 mg/dL (>11.1 mmol/L)	• Overnight fasting, low carbohydrate meal, and exercise are always the first step • Start or maximize metformin • Add SGLT2i or other secondary antihyperglycemia medication (Table 1)	• If FSBG values remain >200 mg/dL after 1 week on metformin and SGLT2i, add an SU or meglitinide (counsel patients on symptoms of hypoglycemia) o Alternatively, consider changing to a different second-line antihyperglycemia medication^a^ o Consider additional carbohydrate restriction to <60 g/day	• If FPG does not resolve to ≤ grade 1 within 21 days after appropriate treatment with antihyperglycemia medication, reduce alpelisib by 1 dose level
**Grade 3:** FPG or fasting FSBG >250–500 mg/dL (>13.9–27.8 mmol/L) or random BG/FSBG consistently >300 mg/dL (>16.7 mmol/L) **OR persistent grade 2**	• Overnight fasting, low carbohydrate meal, and exercise are always the first step • Start/maximize metformin; add/continue SGLT2i or other secondary antihyperglycemia medication; add/continue tertiary SU/meglitinide (Table 1)	• If grade 3 hyperglycemia persists, start SC insulin^a^, either: o Once-daily basal insulin at 0.1–0.2 units/kg SC per day and increase to achieve goal of fasting FSBG <160 mg/dL, or o Prandial rapid-acting insulin sliding scale before meals (do not initiate/continue SU/meglitinide)	• Following appropriate antihyperglycemia treatment: o If FPG ≤ grade 1 within 3–5 days, resume alpelisib at 1 lower dose level o If FPG not ≤ grade 1 within 3–5 days, consult an endocrinologist o If FPG not ≤ grade 1 within 21 days, permanently discontinue alpelisib
**Grade 4:** FPG or fasting FSBG >500 mg/dL (>27.8 mmol/L) or random BG/FSBG consistently >400 mg/dL (>22.2 mmol/L) **OR persistent grade 3**	• Overnight fasting, low carbohydrate meal, and exercise are always the first step • Start/maximize metformin; add/continue SGLT2i or other secondary antihyperglycemia medication; add/continue tertiary SU/meglitinide (Table 1)	• If grade 4 hyperglycemia persists, start SC insulin^a^, either: o Once-daily basal insulin at 0.1–0.2 units/kg SC per day and increase to achieve goal of fasting FSBG <160 mg/dL, or o Prandial rapid-acting insulin sliding scale before meals (do not initiate/continue SU/meglitinide)	• Recheck FPG within 24 h o If FPG is confirmed at grade 4 and confounding factors could be excluded, permanently discontinue alpelisib

BG, blood glucose; BID, twice daily; CTCAE, Common Terminology Criteria for Adverse Events; FPG, fasting plasma glucose; FSBG, fingerstick blood glucose; GA, glycated albumin; OD, once daily; SC, subcutaneous; SGLT2i, sodium-glucose co-transporter 2 inhibitor; SU, sulfonylurea.

a Refer to Table 1 for recommended secondary or tertiary antihyperglycemia medications and dosages, and insulin regimens.

Other second- and third-line antihyperglycemia agents that can be used with metformin are listed in Table 1. However, limited data are available regarding the efficacy of these agents in managing alpelisib-induced diabetes. Counsel patients on symptoms of hypoglycemia if they are taking antihyperglycemia medications that can cause hypoglycemia. Insulin is only recommended for difficult-to-manage grade ≥3 hyperglycemia, since insulin activates the PI3K pathway and has been shown to induce breast cancer cell proliferation in vitro [8,59]. Basal insulin controls glucose levels during periods of fasting [60]. Insulin can be initiated at a dose of 0.1–0.2 units/kg/day and titrated based on clinical response according to the American Diabetes Association guidelines. If prandial insulin is needed, start at the meal with the greatest postprandial excursion [12].

In patients with or at high-risk of developing severe COVID-19, certain oral glucose-lowering medications such as metformin, SGLT2is, thiazolidinediones, and sulfonylureas may need to be discontinued to prevent complications or worsening disease [61].

### 2.2 Skin AEs

#### 2.2.1 Etiology and incidence

**Fig. 2:**
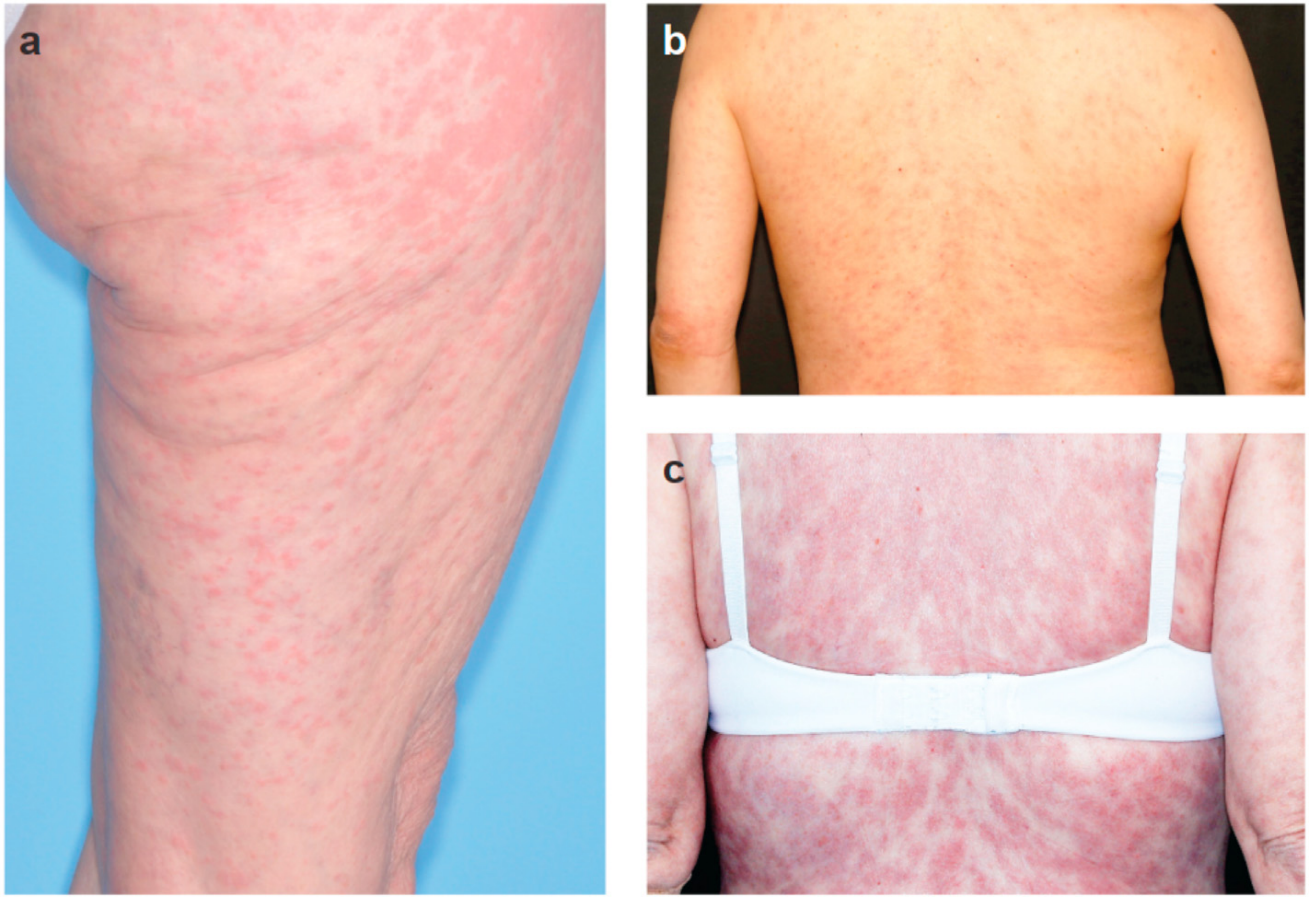
Examples of maculopapular rash in the lower extremity [a], and back [b, c] in breast cancer patients treated with alpelisib. All photos are provided courtesy of Mario E. Lacouture, MD, Dermatology Service, Department of Medicine, Memorial Sloan-Kettering Cancer Center, New York (November 25, 2020).

Skin AEs, particularly the development of rash, are a class effect of PI3Kis [62]. Some of the most frequent skin AEs associated with PI3K inhibition are maculopapular rash, pruritus, and dry skin (Fig. 2) [[62], [63], [64]]. Histamine-producing cells and eosinophils may play a role in PI3Ki-associated rash. Alpelisib-treated patients who developed rash after 2 weeks of treatment showed a significant increase in percentage of blood eosinophils, which may be an indicator of rash formation [65]. In SOLAR-1, the 86 patients who received rash prophylaxis (69.8% received antihistamines) had a lower incidence of all-grade (27% vs 54%) and grade 3 rash (12% vs 20%) compared with the overall population [10,39]. Histologic findings observed with PI3Ki rash are characterized by a perivascular lymphocytic inflammation or dermal hypersensitivity reaction [64]. Rash frequently appears in the torso or extremities [65]. In SOLAR-1, all-grade, grade 3 and all-grade maculopapular rash were reported in 101 (35.6%), 28 (9.9%) and 40 (14.1%) alpelisib-treated patients [8]. The median time to onset of alpelisib-associated rash was 12 days (grade 2/3 rash = 12 days in SOLAR-1, all-grade rash = 12.8 days in a retrospective analysis) [10,65].

#### 2.2.2 Prevention and management

**Fig. 3:**
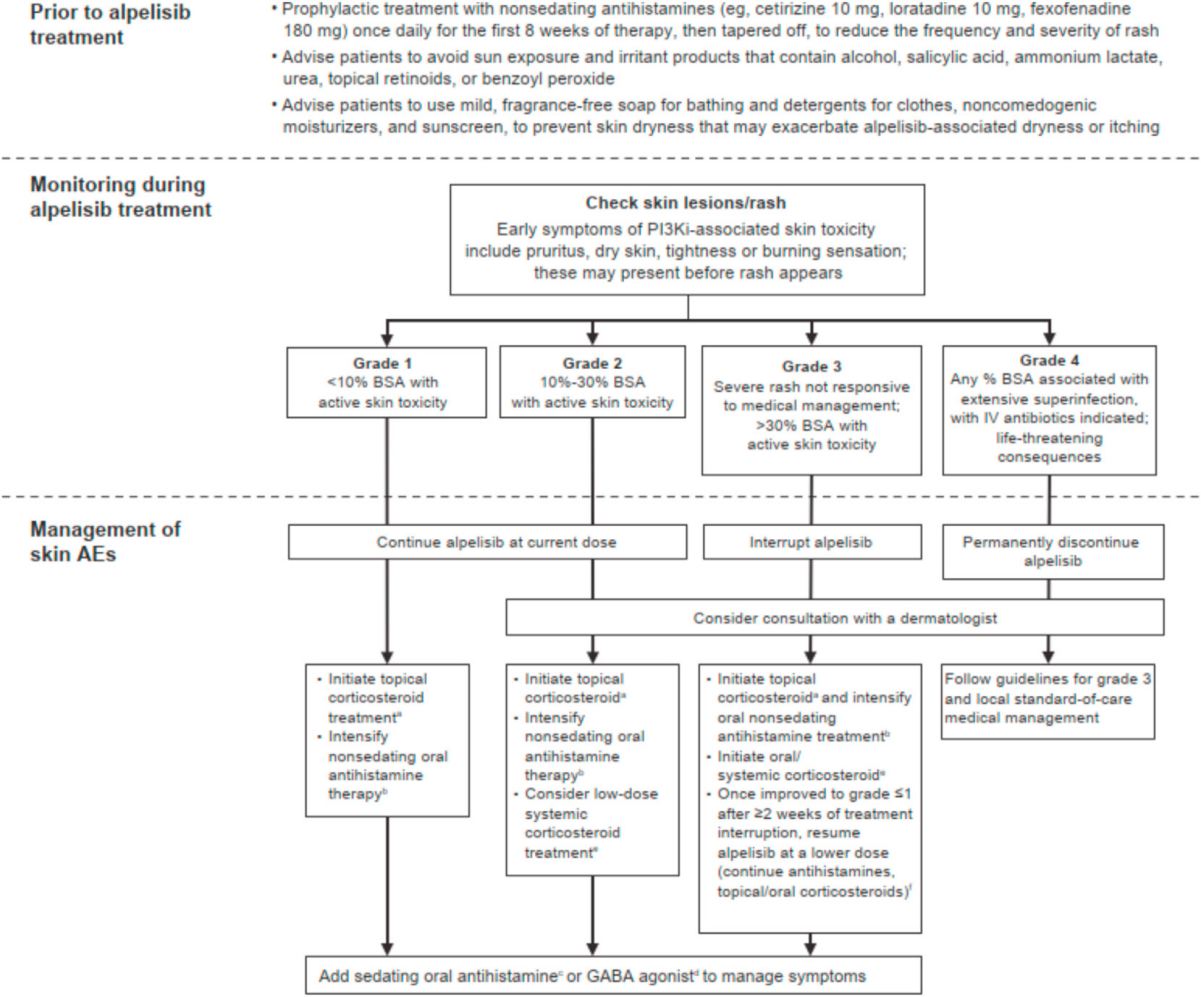
Management of alpelisib-associated skin AEs (severity based on CTCAE v5.0) [66,70]. AE, adverse event; BID, twice daily; BSA, body surface area; CTCAE, Common Terminology Criteria for Adverse Events; GABA, gamma-aminobutyric acid; IV, intravenous; PI3Ki, phosphatidylinositol-3-kinase inhibitor; TID, thrice daily. ^a^Triamcinolone 0.1% or fluocinonide 0.05% BID for ≥28 days. ^b^Cetirizine 10 mg or loratadine 10 mg or fexofenadine 180 mg BID. ^c^Diphenhydramine 25–50 mg or hydroxyzine 25 mg at bedtime. ^d^Gabapentin 300 mg TID or pregabalin 50 mg BID. ^e^Prednisone 0.5–1 mg/kg/daily or equivalent for 7–10 days for grade 3 rash. ^f^If feasible, consider a graded rechallenge with alpelisib starting at 50 mg/day, increasing by 50 mg/week until a 250-mg dose is achieved; start trimethoprim/sulfamethoxazole 160 mg/800 mg three times a week plus a proton pump inhibitor during graded rechallenge (continue oral corticosteroid).

Prophylactic antihistamines should be recommended to all patients starting alpelisib. Initiate nonsedating antihistamines such as cetirizine 10 mg, loratadine 10 mg, or fexofenadine 180 mg prophylactically once daily during the first 8 weeks of therapy then taper off (as onset of rash is likely to occur by 8 weeks) to attempt to reduce the frequency and severity of rash (Table 3 [[66], [67], [68], [69]], Fig. 3 [66,70]). Restart antihistamine therapy at first evidence of skin toxicity thereafter. As antihistamines appear to be less effective once rash occurs, they are recommended primarily for prophylactic use.

**Table 3:** Regimens and characteristics of antirash agents.

Class	Drug and Recommended Dosage	Other Considerations for Treatment
**Sunscreen**	• Broad-spectrum mineral sunscreen, SPF 15 or higher; apply to face and other exposed areas [66]	• SPF/UVA-PF should depend on patient's phototype and induced photosensitivity [66]
**Nonsedating antihistamines**	• Cetirizine 10 mg OD (prophylaxis); 10 mg BID (grade ≥1 rash) • Loratadine 10 mg OD (prophylaxis); 10 mg BID (grade ≥1 rash) • Fexofenadine 180 mg OD (prophylaxis); 180 mg BID (grade ≥1 rash)	• Risk of urinary retention, vasodilation, constipation, blurred vision, dry eyes, reduced motor skills [67]
**Sedating antihistamines**	• Diphenhydramine 25–50 mg at bedtime • Hydroxyzine 25 mg at bedtime	• Risk of CNS depression, photosensitization, urinary retention [67] • Contraindicated in patients with acute asthma exacerbation, narrow-angle glaucoma, GI obstruction, and BPH [67]
**Topical corticosteroids**	• Triamcinolone 0.1% cream or lotion BID • Fluocinonide 0.05% cream or lotion BID	• Risk for skin atrophy, striae, rosacea, perioral dermatitis, acne, purpura with long-term use (>12 weeks) [68] • Less common reactions include hypertrichosis, change in skin pigmentation, delayed wound healing, exacerbation of skin infections [68] • May rarely lead to hyperglycemia, glaucoma, and adrenal insufficiency [68]
**Oral corticosteroids**	• Prednisone 0.5–1 mg/kg/daily or equivalent	• Risk of hyperglycemia, immunosuppression, osteoporosis/fractures, weight gain, CVD, dyslipidemia, myopathy, cataracts, glaucoma, psychiatric disturbances, HPA-axis suppression [69]
**GABA agonist**	• Gabapentin 300 mg TID • Pregabalin 50 mg BID	• Risk of somnolence, dizziness, peripheral edema (gabapentin)

BID, twice daily; BPH, benign prostatic hypertrophy; CNS, central nervous system; CVD, cardiovascular disease; GABA, gamma-aminobutyric acid; HPA, hypothalamic-pituitary-adrenal; GI, gastrointestinal; OD, once daily; SPF, sun protection factor; TID, thrice daily; UVA-PF, ultraviolet A protection factor.

Patients should be advised to avoid unprotected sun exposure and irritant products that contain alcohol, salicylic acid, ammonium lactate, urea, topical retinoids, or benzoyl peroxide [66]. Advise patients to use a mild, fragrance-free soap for bathing and detergents for clothes, noncomedogenic moisturizers, and sunscreen to prevent skin dryness that may exacerbate alpelisib-associated dryness or itching [8,66].

Most skin reactions are reversible with adequate co-medication and treatment interruption. Treat patients symptomatically and according to clinical severity. Clinicians may consider increasing doses of cetirizine, loratadine, or fexofenadine to BID for grade ≥1 rash—however, there is currently no evidence to support that this decreases rash. Initiate topical corticosteroids (triamcinolone 0.1% or fluocinonide 0.05%) BID for ≥28 days for grades 1/2 rash (Fig. 3) [66,70]. Consider the use of lotion or solution preparations for the trunk and the scalp, for ease of application. For burning, stinging, or pruritus, consider adding sedating antihistamines at bedtime (diphenhydramine 25–50 mg or hydroxyzine 25 mg) or gamma-aminobutyric acid agonists (gabapentin 300 mg thrice daily [TID] or pregabalin 50 mg BID) (Table 3) [[66], [67], [68], [69]].

For grade 3 rash, interrupt alpelisib, intensify oral antihistamines, and start a 7- to 10-day course of oral systemic corticosteroids (prednisone 0.5–1 mg/kg/day or equivalent). Rechallenge with alpelisib after ≥2 weeks’ treatment interruption and upon resolution to grade ≤1 rash (continue antihistamines and oral corticosteroids). Consult dermatologists for refractory grade 2 or any grade ≥3 skin AEs. Take caution with use of systemic steroids as these may worsen hyperglycemia [71]. If feasible, consider a graded rechallenge in patients with recurrence of grade 3 rash who demonstrate improvement to grade ≤1 following treatment interruption.

In a real-world study, 2 of 8 patients treated with alpelisib experienced rash. In 1 patient with grade 3 rash, rash was managed with alpelisib treatment interruption, antihistamine and cortisone therapy. In the other patient (grade 1 rash), no treatment interruption was necessary, and rash was effectively managed with cortisone and antihistamines [72].

#### 2.2.3 Serious cutaneous reactions

Serious cutaneous reactions with alpelisib appear to be rare; Stevens-Johnson syndrome and erythema multiforme were reported in 0.4% and 1.1% of patients, respectively [8]. Drug reaction with eosinophilia and systemic symptoms was reported in alpelisib-treated patients in the postmarketing setting [10]. Prompt identification and discontinuation of alpelisib are critical. Supportive care is the mainstay of management, which includes wound care; maintaining adequate hydration, nutrition, and electrolyte balance; maintaining renal and airway function; and pain management [73]. If a serious reaction is confirmed, permanently discontinue alpelisib. Do not reintroduce alpelisib in patients who experienced previous serious cutaneous reactions such as Stevens-Johnson syndrome with alpelisib [10].

### 2.3 Diarrhea

#### 2.3.1 Etiology and incidence

Diarrhea, with or without colitis, is an immune-mediated reaction associated with PI3Kis [74]. Overall, 58% (n = 164) of alpelisib-treated patients in SOLAR-1 experienced diarrhea, with a median time to onset of grade 2/3 diarrhea of 46 days [8,10]. Grade 3 diarrhea was reported in 7% (n = 19) of patients [8]. Median time to improvement by ≥ 1 grade for grade ≥3 diarrhea was 18 days [39].

#### 2.3.2 Prevention and management

Before starting alpelisib, review patients’ medical histories to identify those with history of chronic diarrhea, or those with diarrhea-inducing diseases or conditions. Discontinue any diarrheagenic agents at screening. In patients presenting with recent antibiotic intake and loose stools, rule out *Clostridium difficile* infection.

In patients presenting with alpelisib-associated diarrhea, monitor frequency of bowel movements and advise patients to drink 8–10 glasses of clear liquids/day (eg, water, oral electrolyte solution, broth) and to eat frequent, small meals, which may include banana, rice, applesauce, and toast. Recommend loperamide (2 mg/dose, 2 doses after first loose stool and 1 dose after each subsequent watery stool, taken every 4 h) or diphenoxylate hydrochloride/atropine sulfate. Diphenoxylate hydrochloride/atropine sulfate should not be used in combination with loperamide due to the risk of paralytic ileus. After a 12-h diarrhea-free interval, loperamide may be discontinued. If diarrhea persists (≥4 episodes despite use of loperamide) or the patient cannot maintain hydration, advise the patient to seek treatment urgently. For grade 2 diarrhea (increase of 4–6 stools/day or moderate increase in ostomy output over baseline), interrupt alpelisib until recovery to grade ≤1, then resume at same dose level.

For grade 3–4 diarrhea (increase of ≥7 stools/day or severe increase in ostomy output over baseline, hospitalization indicated, limiting self-care activities of daily living, or with life-threatening consequences), interrupt alpelisib until recovery to grade ≤1, then resume alpelisib at next dose level. Consider hospital admission for intravenous (IV) hydration and antibiotics. Increase loperamide frequency to every 2 h, for up to a maximum of 16 mg/day. Additional antidiarrheal medications such as opium tincture or dihydrocodeine tartrate oral/subcutaneous [SC]/intramuscular [IM] may be given; administer Sandostatin/octreotide 100–500 μg TID SC if severe diarrhea persists after 12–24 h, then 500–1000 μg TID SC if severe diarrhea still persists. Work-up for severe diarrhea may include stool analysis for blood, fecal leukocytes (Wright's staining and microscopy) or *Clostridium difficile* toxin, and fecal cultures for *Salmonella* spp., *Campylobacter* spp., *Giardia*, *Entamoeba*, *Cryptosporidium*, *Shigella*, and pathogenic *E. coli.*

### 2.4 Other alpelisib-associated AEs

Another common AE observed in alpelisib-treated patients (n = 284) is stomatitis (24.6%) [8]. At the first sign of aphthous stomatitis, initiate dexamethasone mouth rinse (swish and spit) TID for 4–6 weeks, and use only as needed thereafter. Another treatment for stomatitis is steroid (eg, triamcinolone) dental paste. Instruct patients to apply 2–4 times a day on affected areas until the lesion is healed [75]. For other AEs such as nausea and vomiting (SOLAR-1 incidences were 44.7% and 27.1%, respectively) [8], initiate appropriate medical therapy and monitor as clinically indicated.

Although rare, cases of pneumonitis (n = 2, 0.7%) and pancreatitis (n = 1, 0.4%) were observed in alpelisib-treated patients [8]. For suspected cases of pneumonitis, obtain appropriate imaging such as a high-resolution chest computed tomography scan to confirm diagnosis and rule out infectious causes of interstitial lung disease. Consider hospital admission, broncho-alveolar lavage, and biopsy. High-dose corticosteroids and consultation with a pulmonologist should be recommended, and antibiotic therapy initiated if infectious causes are suspected [8,76].

## 3 Conclusions

Alpelisib is associated with a complex set of AEs that if recognized early and managed appropriately could be effectively controlled, thus allowing patients to continue treatment longer. Discontinuation rates due to any-grade AE (29.2% vs 20.7%) and grade ≥3 (18.1% vs 7.9%) were lower in the second compared with the first half of randomized patients in the alpelisib group in SOLAR-1 with amending the protocol to restrict eligibility to patients with HbA1c ≤ 6.5% and with increased physician experience managing toxicities [8,39]. Multidisciplinary involvement and patient education are critical (see Appendix A for a plain-language summary of this manuscript). Clinicians should also consider COVID-19–associated risks and complications [77,78].

BYLieve is an ongoing phase 2 study evaluating the safety and efficacy of alpelisib plus endocrine therapy in a post–cyclin-dependent kinase 4/6 inhibitor setting. The most common AEs reported in the fulvestrant (n = 127) and letrozole (n = 126) cohorts were consistent with SOLAR-1 [79,80]. However, compared with SOLAR-1, discontinuation rates for hyperglycemia (SOLAR-1 n = 18 [6.3%] vs BYLieve fulvestrant cohort n = 2 [2%] and letrozole cohort n = 1 [0.8%]) were lower in the BYLieve trial in both cohorts [8,79,80]. Therefore, it is possible that greater experience with hyperglycemia and eventually other PI3Ki-related toxicities will reduce their severity.

Further studies to explore risk factors and the pathogenesis of the AEs associated with alpha-selective PI3K-inhibition are needed to continually improve patient outcomes.

## Declarations

### Role of the funding source

Medical editorial assistance was provided by Audrey Clement So, MD, from Healthcare Consultancy Group, LLC, and was funded by 10.13039/100008272Novartis Pharmaceuticals Corporation. M.E. Lacouture is supported in part by the 10.13039/100000002NIH / 10.13039/100000054NCI 10.13039/100007345Cancer Center Support Grant P30 CA008748.

## Author contributions

All authors contributed equally to the conceptualization of this review. All authors reviewed the literature and each provided their insights and expert recommendations. This draft has been developed and approved for submission by all authors. All persons listed as authors have contributed to preparing the manuscript and the International Committee of Medical Journal Editors (ICMJE) criteria for authorship have been met.

## Declaration of competing interest

**H.S. Rugo:** Institution research funding from Pfizer, Novartis, Eli Lilly, Genentech, MacroGenics, Merck, OBI Pharma, Eisai, Immunomedics, Daiichi, Odonate, and Seattle Genetics; travel support from Daiichi, AstraZeneca, Novartis, Pfizer, Mylan, and Merck; consulting/advisory income from Puma and Celltrion. **M.E. Lacouture:** Consulting from 10.13039/100004336Novartis, Varsona, Innovaderm, 10.13039/100012634Novocure, QED, Seagen, Lutris, DFB, JnJ, Deciphera, Onquality, TWIBiotech, Azitra, Janssen, 10.13039/100004755EMD Serono, and Bicara; research funding from 10.13039/100004336Novartis, AZ, JnJ, Onquality, and 10.13039/100012634Novocure. **M.D. Goncalves:** Personal fees from 10.13039/100004336Novartis, grants from 10.13039/100004319Pfizer, and personal fees from Petra Pharma, Scorpion Therapeutics, and Faeth Therapeutics, outside the submitted work; patent (pending) for Combination Therapy for PI3K-associated Disease or Disorder, and patent for The Identification of Therapeutic Interventions to Improve Response to PI3K Inhibitors for Cancer Treatment pending; and owns stock in Faeth Therapeutics. **U. Masharani:** Research funding from Clementia Pharmaceuticals. **M.S. Aapro:** Consulting/advisory role in 10.13039/100002429Amgen, 10.13039/100002491BMS, Daiichi Sankyo, Fresenius Kabi, G1 Therapeutics, 10.13039/100008067Genomic Health, 10.13039/100008129Helsinn Healthcare, 10.13039/100004334Merck, 10.13039/100009945Merck KGaA, 10.13039/100004336Novartis, 10.13039/100004319Pfizer, 10.13039/100013226Pierre Fabre, 10.13039/100004337Roche, 10.13039/100011218Sandoz, Tesaro, and Vifor Pharma; speakers' bureau at Accord Research, 10.13039/100002429Amgen, 10.13039/100007777Biocon, Dr Reed, 10.13039/100008067Genomic Health, 10.13039/100008129Helsinn Healthcare, Mundipharma, 10.13039/100004336Novartis, 10.13039/100004319Pfizer, 10.13039/100013226Pierre Fabre, 10.13039/100004337Roche, 10.13039/100011218Sandoz, 10.13039/100009954Taiho Pharmaceutical, Tesaro, and Vifor Pharma; research funding from 10.13039/100008129Helsinn Healthcare, 10.13039/100004336Novartis, 10.13039/100013226Pierre Fabre, and 10.13039/100011218Sandoz. **J.A. O'Shaughnessy:** Personal fees from 10.13039/100006483AbbVie, Agendia, 10.13039/100002429Amgen, 10.13039/100004325AstraZeneca, 10.13039/100002491Bristol-Myers Squibb, 10.13039/100006436Celgene, 10.13039/501100003769Eisai, 10.13039/100004328Genentech, 10.13039/100008067Genomic Health, GRAIL, Immunomedics, Heron Therapeutics, 10.13039/100013733Ipsen Biopharmaceuticals, Jounce Therapeutics, 10.13039/100004312Lilly, 10.13039/100004334Merck, Myriad, 10.13039/100004336Novartis, Ondonate Therapeutics, 10.13039/100004319Pfizer, Puma Biotechnology, Prime Oncology, 10.13039/100004337Roche, 10.13039/100010293Seattle Genetics, and Syndax Pharmaceuticals, outside the submitted work.
